# Inter-Fighting between Influenza A Virus NS1 and β-TrCP: A Novel Mechanism of Anti-Influenza Virus

**DOI:** 10.3390/v14112426

**Published:** 2022-10-31

**Authors:** Haiwei Sun, Kai Wang, Wei Yao, Jingyi Liu, Lu Lv, Xinjin Shi, Hongjun Chen

**Affiliations:** 1 Shanghai Veterinary Research Institute, Chinese Academy of Agricultural Sciences, Shanghai 200241, China; 2 Biosafety Research Center, Chinese Academy of Agricultural Sciences, Shanghai 200241, China

**Keywords:** influenza A virus, NS1, β-TrCP, replication

## Abstract

Influenza A virus (IAV) prevents innate immune signaling during infection. In our previous study, the production of pro-inflammatory cytokines was associated with Cullin-1 RING ligase (CRL1), which was related to NF-κB activation. However, the underlying mechanism is unclear. Here, an E3 ligase, β-transducin repeat-containing protein (β-TrCP), was significantly downregulated during IAV infection. Co-IP analysis revealed that non-structural 1 protein (NS1) interacts with β-TrCP. With co-transfection, an increase in NS1 expression led to a reduction in β-TrCP expression, affecting the level of IκBα and then resulting in repression of the activation of the NF-κB pathway during IAV infection. In addition, β-TrCP targets the viral NS1 protein and significantly reduces the replication level of influenza virus. Our results provide a novel mechanism for influenza to modulate its immune response during infection, and β-TrCP may be a novel target for influenza virus antagonism.

## 1. Introduction

In the past few years, research into viruses’ evasion host defenses has received increasing attention because of the mechanisms that would help to develop attenuated vaccines or antiviral therapies targeting specific immune system antagonists [1,2,3]. Most, if not all, viruses encode proteins that are involved in the induction of adhesion molecules, accelerating inflammation and diapedesis of effector cells of the innate immunity, particularly the innate immune responses driven by the transcription factors of NF-κB, IRF-3, and AP-1 [2,4,5,6].

IAVs, the major causes of respiratory illness in humans and animals, are RNA viruses that belong to the family *Orthomyxoviridae* [7], which trigger the activation of innate immunity and adaptive immunity to exert antiviral capabilities. Meanwhile, IAVs have evolved different strategies to minimize the activation of antiviral capabilities. IAV mainly suppresses the host antiviral immune response by inhibiting IFN production and ISG antiviral activity while viral NS1 is mainly responsible for antagonizing the IFN antiviral response.

IAVs usually cause acute respiratory inflammation [8,9,10]. The symptoms include a high fever, body aches, and fatigue. Severe influenza infection often causes high morbidity and mortality due to an overly aggressive innate immune response with early recruitment of inflammatory leukocytes to the lung microenvironment [5,8,11,12]. For example, the high pathogenic Influenza A virus H5N1 and 2009 pandemic H1N1 caused many deaths, which are reported to be associated with excessive pro-inflammatory production and the cytokine storm induced by influenza infection [4,13,14,15]. Generally, the immune response directly caused by influenza viruses is a sprawling network amplified by autocrine and paracrine mediator cascades. Pathways associated with PRRs, ILs, IFN-I, TNFs, cyclooxygenase (COX)-2, and c-Jun N-terminal kinase (JNK) are activated in the transcription of NF-κB and the formation of inflammasomes. However, the critical factors in this network, especially those specific to the pathogenesis of severe influenza, are still unknown [16].

In our previous study, we found that the expression of pro-inflammatory cytokines (IL-1β, IL-6, and TNF-α) was dramatically increased in IAV-infected CUL1 knock-down A549 cells while the use of MLN4924 to block CULs neddylation significantly suppressed the expression of pro-inflammatory cytokines induced by IAVs [17]. β-TrCP is a well-characterized mammalian F-box protein of Skp1-CUL1-F-box (SCF) ligase [6,16,18,19]. In the SCF^β-TrCP^ complex, Skp1 functions as an adaptor that binds to the N-terminus of CUL1 via its N-terminus and β-TrCP via its C-terminus while the RING protein RBX1 binds to the C-terminus of CUL1 via its N-terminus and ubiquitin E2 via its C-terminus. β-TrCP recognizes the substrates that contain the DSGxxS degron motif or its variants. Meanwhile, the ubiquitin could be effectively transferred from RBX1-bound ubiquitin-charged E2 to degrade the substrate [16]. SCF^β-TrCP^ ubiquitin ligase was demonstrated to degrade critical regulators involved in different biological functions, such as signal transduction, cell cycle, apoptosis, and virus infection [20]. In addition, SCF^β-TrCP^ ubiquitin ligase plays a vital role in regulating immune responses [20]. Several immune-related proteins such as IκB, TNFAIP8-like 2 (TIPE2), and interleukin 1 receptor-associated kinase 1 (IRAK1) were recognized by β-TrCP and further degraded by ubiquitin [21,22,23]. Degradation of IκB by β-TrCP would promote the translocation of NF-κB to the nucleus, inducing the expression of inflammatory cytokines.

In contrast, evidence shows that β-TrCP could be ubiquitinated and degraded by the proteasome. For example, Rotavirus NSP1 protein hijacks CUL3-Rbx1 complex to target β-TrCP degradation [24]. IAV utilizes various strategies to suppress host innate immune responses, facilitating viral replication in host cells. In this study, we hypothesized that IAV targets β-TrCP to inhibit the degradation of IκB, negatively regulating the induction of pro-inflammatory cytokines via the NF-κB signaling pathway. Given the importance of inflammatory reactions, the activity and stability of β-TrCP should be strictly controlled.

However, the potential role of β-TrCP in the regulation of the pro-inflammatory responses and the mechanisms of the SCF^β-TrCP^-activating NF-κB pathway induced by influenza viruses have not been well defined. It is essential to determine the expression patterns of the SCF^β-TrCP^/NF-κB pathway in influenza A virus (IAV) infections. This would be helpful for understanding the mechanisms of the immune responses in influenza A virus infections.

## 2. Materials and Methods

### 2.1. Cell Culture

The human alveolar type II epithelial cell line A549 (ATCC CCL-185), Madin–Darby canine kidney epithelial (MDCK) cell line (ATCC CCL-34), and human embryonic kidney 293T cell line (ATCC CCL-3216) were grown in Dulbecco’ s Modified Eagle’s medium (DMEM) (Thermo Fisher Scientific Inc., Waltham, MA, USA) supplemented with 5% fetal bovine serum (FBS) and 1% Penicillin-Streptomycin solution (Thermo) at 37 °C with 5% CO_2_.

### 2.2. Construction of Expression Plasmids and Transient Transfection

cDNA encoding full-length human β-TrCP were subcloned in-frame mammalian expression vector pcDNA3 (Invitrogen), with an N-terminal Flag, HA-tag, or Myc-tag. NS1 of IAVs were subcloned into pcDNA3 (Invitrogen) with an N-terminal Flag-tag, HA-tag, or Myc-tag.

### 2.3. Viruses

Mouse-adapted A/California/04/09 (H1N1) (maCa04) was a mutated virus of maCa04 blindly passaged into DBA mice, a gift from Dr. Daniel R. Perez [25]. A/Puerto Rico/8/34 (H1N1) (PR8) was amplified in 9-day-old SPF embryo eggs at 37 °C for 48 h. The allantoic fluid was collected in vials and stored at −80 °C until use. The viral titers were determined and calculated according to the Reed–Muench method for a 50% tissue culture infectious dose (TCID_50_) on MDCK cells with MEM with 2% bovine serum albumin (BSA) and 1 μg/mL trypsin treated with L-(tosylamido-2-phenyl) ethyl chloromethyl ketone (TPCK).

### 2.4. Virus Infections and Quantitation of Viral Loads

Cells (1 × 10^6^/well) were passaged into a 100-mm dish with 90–95% confluence overnight. The cells were infected with IAVs at a multiplicity of infection (m.o.i) of 1 and incubated at 37 °C for 2 h. After incubation, the viral supernatants were removed and replaced with fresh culture media. The cells were collected at the indicated time points to determine the viral loads by immunoblot analysis.

### 2.5. Knock-Down of β-TrCP Expression

siRNA oligonucleotides against β-TrCP and a nontargeting control siRNA were purchased from Genepharma. For siRNA gene knock-down experiments, the cells were cultured in 6-well plates, and the 40 nM siRNA solution was transfected with lipofectamine 2000 (Invitrogen) according to the manufacturer’s instructions. After 48 h of transfection, the cells were analyzed to determine the knockdown efficiency. The β-TrCP RNA interference target sequences were as follows: si-β-TrCP-1#: GUGCCAGACUCUGCUUAAATT, and si-β-TrCP-2#: GGGACAGUAUUUAUUCAAATT. The control siRNA sequence was UUCUCCGAACGUGUCACGUTT.

### 2.6. Immunoblot Analysis

The immunoblot experiment was carried out as previously described [17]. Briefly, cells were washed with phosphate-buffered saline (PBS), lysed with a mammalian lysis buffer (50 mM Tris-Cl, pH 8.0, 150 mM NaCl, 1 mM EDTA, 1% Nonidet P-40, and 0.4 mM phenylmethylsulfonyl fluoride (PMSF)) and sonicated, then denatured by incubation for 5 min at 95 °C in a sample buffer (2% SDS, 10% glycerol, 60 mM Tris (pH 6.8), 5% β-mercaptoethanol, and 0.01% bromophenol blue). After that, the samples were subjected to sodium dodecyl sulfate-polyacrylamide gel electrophoresis (SDS-PAGE) and then transferred to the nitrocellulose transfer membrane (PALL Shanghai, China) using the Pyxis^TM^ Gel Processor. After being blocked in 0.05% Tween-PBS with 5% skim milk for 30 min, the membranes were subjected to immunoblot with the primary antibodies and subsequently with HRP-conjugated secondary antibodies. After washing with PBST (PBS buffer containing 0.05% Tween 20) for 30 min, the membranes were then developed with ECL (Thermo) and imaged by the Tannon 5200 system (Tannon Inc., Shanghai, China). Antibodies were used, including mouse anti-CUL1 antibody (1:1000, Santa Cruz Biotechnology, Santa Cruz, CA, USA), rabbit anti-β-TrCP antibody (1:1000, Cell Signaling, Danvers, MA, USA), goat anti-PB2 antibody (1:500, Santa Cruz), goat anti-PB1 antibody (1:500, Santa Cruz), rabbit anti-PA antibody (1:2000, Gene Tex, Irvine, CA, USA), rabbit anti-NP antibody (1:2000, Gene Tex), rabbit anti-NA antibody (1:1000, Thermo, USA), rabbit anti-NS1 antibody (1:2000, Gene Tex), rabbit anti-M1 antibody (1:1000, Thermo, USA), rabbit anti-HA tag antibody (1:2000, Cell Signaling), rabbit anti-Flag antibody (1:2000, Cell Signaling) or mouse anti-Flag antibody (1:2000, Millipore Sigma, St. Louis, MO, USA), mouse anti-Myc antibody (1:2000, Sigma), rabbit anti-IκBα antibody (1:1000, Cell Signaling Technology, USA), and mouse anti β-actin antibody (1:1000, Cell Signaling). Secondary antibodies included HRP-conjugated goat anti-rabbit IgG antibody and anti-mouse IgG antibody (1:2000, Southern Biotech, Birmingham, AL, USA), HRP-conjugated donkey anti-rabbit IgG (H+L) antibody (1:2000, Santa Cruz), HRP-conjugated mouse anti-rabbit IgG antibody (1:1000, Abbkine, Wuhan, China), and HRP-conjugated goat anti-rabbit IgG antibody (1:1000, Abbkine).

### 2.7. Immunoprecipitation Analysis

293T cells were collected and lysed in RIPA lysis buffer (Yeasen, Shanghai, China) supplemented with protease inhibitor Cocktail (Yeasen) for 30 min at 4 °C, centrifuged at 8800 rpm for 10 min, the supernatant was collected and divided into three 1.5 mL tube (one for input, one for IgG, and one for IP). For the immunoprecipitation assay, the cell lysate supernatants were incubated for 4 h at 4 °C with anti-Flag (Sigma) or anti-HA (Cell Signaling) coupled to the protein A/G-agarose beads (Sigma). Then, immunoprecipitated beads were washed 5–6 times with RIPA lysis buffer. Immunoprecipitates and input were subjected to run SDS-PAGE gel, and the next step was the same as the immunoblot assay.

### 2.8. Pro-Inflammatory Cytokine Levels by rRT-PCR

The infected cells were thawed, and the total RNA of 200-μL supernatants was treated and extracted with an RNeasy mini kit (Qiagen Corporation, Hilden, Germany) from the cells above according to the manufacturer’s instructions. The mRNA was primed using Oligo (dT)18 (Promega Co., Madison, WS, USA), and the cDNA synthesis was driven by AMV transcriptase (Promega) in 20-μL volumes at 30 °C for 10 min, 42 °C for 2 h, and then 70 °C for 15 min. The primers of RT-PCR were designed using the PrimerQuest Tool. The sequences of the primers were as follows: β-actin-107F: GGACCTGACTGACTACCTCAT; β-actin-107R: CGTAGCACAG CTTCTCCTTAAT. IL-1β-102F: CATGGGATAACGA GGCTT ATGT; IL-1β-102R: CATATGGACCAGACATCACCAA. IL-6-107F: CACT CACCT CTTCAGAACGAAT; IL-6-107R: GCTGCTTTCACACATGTTACTC. TNF-α-106F: CCAGGGACCTCTCTCTAAT CA; TNF-α-106R: TCAGCTTGAGGGT TTGCTAC. The reactions were conducted by rRT-PCR analysis with AceQ^®^ qPCR SYBR^®^ Green Master Mix (Vazyme Inc., Nanjing, China) according to the following cycle protocol: 95 °C for 5 min, 40 cycles at 95 °C for 10 s, and 60 °C for 30 s or followed by the melt curve stage (95 °C for 15 s, 60 °C for 1 min, and 95 °C for 15 s). The reaction results were represented by threshold cycle (Ct) values. The fold change was calculated with the 2^-ΔΔCt^ method as in previous studies. The mean Ct values were determined based on triplicates.

### 2.9. Statistical Analysis

All the data were graphed, and statistical analyses were performed using Prism 6 software (GraphPad, La Jolla, CA, USA). Comparisons between two groups’ means were performed with a two-tailed Student’s *t*-test, whereas multiple comparisons were conducted by one-way analysis of variance (ANOVA). The differences were considered statistically significant at *p* values of <0.05 or <0.01.

## 3. Results

### 3.1. IAV Infection Induces β-TrCP Degradation

**Figure 1:**
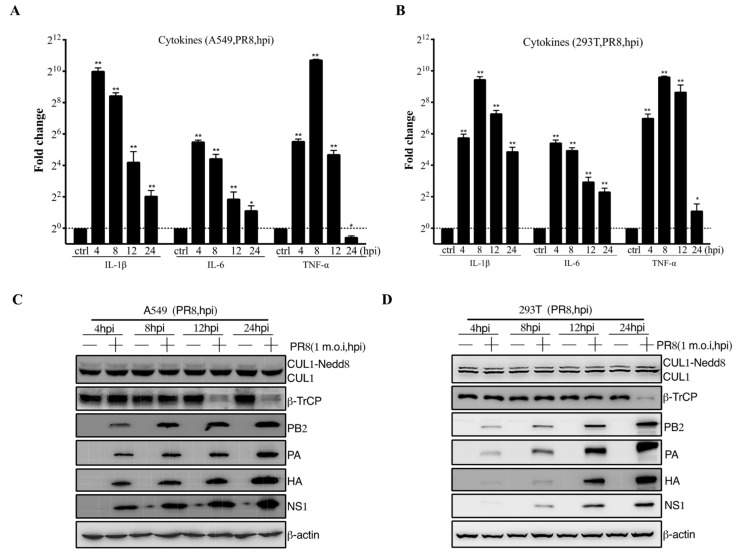
The role of β-TrCP in the process of influenza A virus infection in mammalian cells. A549 and 293T cells were treated with PR8 virus at 1 m.o.i, and the samples were collected within 24 hpi, and the transcriptional changes of IL-1β, IL-6, and TNF-α mRNAs were determined by qRT-PCR (**A**,**B**); and the protein expression changes in CUL1, β-TrCP, viral protein PA, HA, and NS1 were determined by immunoblot analysis, and β-actin as a loading control (**C**,**D**). The data represent the mean values ± SD from three independent experiments in triplicate. *: *p* < 0.05, **: *p* < 0.01.

**Figure 2:**
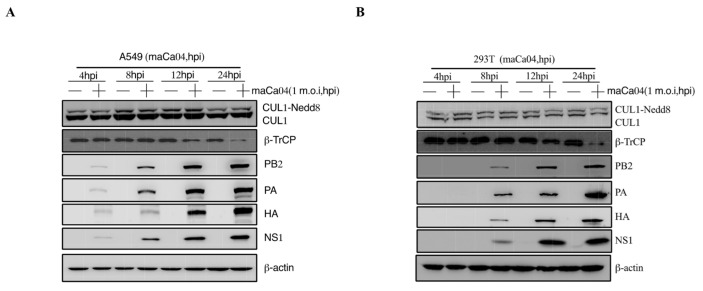
The role of β-TrCP in the process of maCa04 (H1N1) infection in mammalian cells. A549 cells (**A**) and 293T cells (**B**) were treated with maCa04 virus at 1 m.o.i, and the samples were collected within 24 hpi; the protein expression changes in CUL1, β-TrCP, viral protein PA, HA, and NS1 were determined by immunoblot analysis; and β-actin was used as a loading control.

The pathogenicity of influenza viruses was associated with viral self-replication and the inflammatory response induced by IAVs infection [26]. To investigate the inflammatory response of influenza virus infection to host cells, A549 and 293T cells were infected with PR8 virus at 1 m.o.i for 4, 8, 12, and 24 hpi, respectively. Then, the mRNA expression level of proinflammatory cytokines, including IL-1β, IL-6, and TNF-α, were remarkably reduced at 12–24 hpi after PR8 virus infection (Figure 1A,B). Previous studies have shown that CRL1/β-TrCP is critical for polyubiquitination of IκBα [27]. Therefore, the CRL1/β-TrCP pathway may play an important role in regulating the inflammatory response to IAVs infection. To systematically explore the association between CRL1/β-TrCP and IAVs, the PR8-infected A549 and 293T cells were subjected to immunoblot analysis. The results showed that β-TrCP was significantly reduced at 12–24 hpi PR8 virus infection (Figure 1C,D). Meanwhile, the level of viral proteins PB2, PA, HA, and NS1 served as the indicator of IAVs replication in A549 and 293T cells by immunoblot analysis. In addition, to avoid the β-TrCP reduction in PR8-infected cells being viral specific, maCa04 (H1N1 virus) was performed in this study, and our data showed that β-TrCP was also reduced in maCa04-infected A549 cells and 293T cells (Figure 2A,B). Thus, our data indicate that β-TrCP could be an important host protein in influenza virus infection.

### 3.2. Identification of IAV NS1 Interacting with β-TrCP

**Figure 3:**
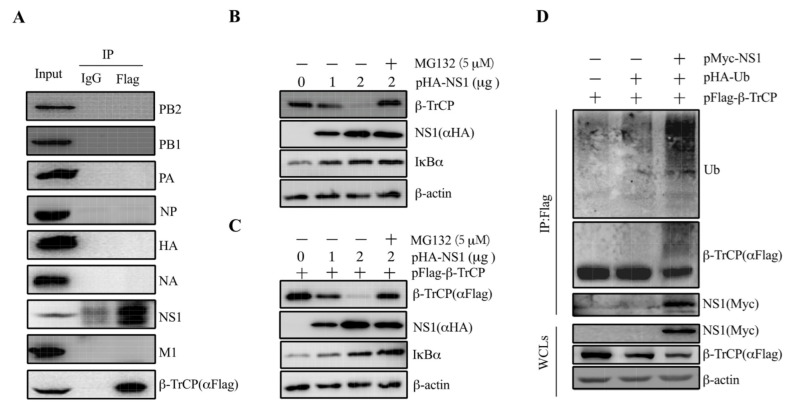
IAV NS1 associates with β-TrCP. 293T cells were transfected with pFlag-β-TrCP and then infected with PR8 virus at 1 m.o.i for 24 h. Then, the cell lysates were harvested and immunoprecipitated with anti-Flag and immunoblotted with anti-Flag, PB2, PB1, PA, NP, HA, NA, NS1, and M1 (**A**). 293T cells were transiently transfected with pHA-NS1 along with (**B**) or without (**C**) pFlag-β-TrCP for 24 h and then incubated for an additional 24 h in the presence or absence of MG132 (5 μM). The levels of pFlag-β-TrCP and pHA-NS1 were measured by anti-Flag and anti-HA, respectively. Endogenous β-TrCP and β-actin levels were measured by their respective antibodies. pFlag-β-TrCP and pHA-Ub were co-transfected in 293T cells with pMyc-tagged empty vector and pPR8-NS1, and the cell lysates were harvested and subjected to immunoprecipitation with anti-Flag and immunoblot with anti-Flag, Myc, and Ub antibodies (**D**).

To illustrate our speculation that β-TrCP might interact with viral proteins, we performed Co-IP assays to screen the β-TrCP-interacting IAV proteins. β-TrCP was fused with Flag tag as a bait protein and transfected into 293T cells, followed by the infection of PR8 at 1 m.o.i. At 24 hpi, the cell lysates were collected for further analysis. As shown, the viral proteins PB2, PB1, PA, NP, HA, NA, and M were not pulled down by pFlag-β-TrCP, except NS1, suggesting that IAV NS1 protein could interact with β-TrCP (Figure 3A). To exclude that the interaction is strain specific, we examined the interaction of NS1 from maCa04 with β-TrCP. The results showed that both maCa04 and PR8 NS1 proteins were associated with β-TrCP (Figure S1), indicating that IAV NS1 protein can physically associate with β-TrCP.

### 3.3. NS1 Mediates IκBα Expression by Targeting β-TrCP

To confirm the interaction between β-TrCP and NS1, 293T cells were transfected with pHA-NS1 alone or co-transfected with pFlag-β-TrCP. The results showed that the increase in NS1 resulted in decreased expression of β-TrCP in a dose-dependent manner in the cells transfected with pHA-NS1 alone (Figure 3B). The degradation of β-TrCP was blocked by the proteasome inhibitor MG132. Similar results were observed in the cells co-transfected with pHA-NS1 and pFlag-β-TrCP (Figure 3C), confirming that NS1 could interact with β-TrCP. These results suggested that NS1 targets β-TrCP for its proteasomal degradation.

IκBα plays an important role in the inhibition of NF-κB activation, which in turn influences the expression of proinflammatory cytokines. Given that β-TrCP was previously reported to activate NF-κB signaling by degrading IκBα expression [27], we investigated whether the expression of NS1 has an impact on the expression of IκBα. As shown in Figure 3B, the decrease in NS1 resulted in an increase in the expression of IκBα in a dose-dependent manner in the cells transfected with pHA-NS1 alone. Similar results were observed in the cells transfected with pHA-NS1 along with pFlag-β-TrCP (Figure 3C), indicating that there might be an association between β-TrCP and IκBα. These results suggest that IAVs could suppress host proinflammatory cytokines’ expression via the degradation of IκBα via viral NS1 protein targeting β-TrCP.

### 3.4. NS1 Promotes β-TrCP Degradation Via Ubiquitination

It is known that β-TrCP degrades a wide range of substrates that affects down-strain signaling pathways [21,22,23]. Interestingly, our data support that NS1 protein targets β-TrCP for its proteasomal degradation. In order to answer this question, 293T cells were co-transfected with pFlag-β-TrCP, pHA-Ub, and pMyc-NS1. The cell lysates were harvested and subjected to immunoprecipitation and immunoblot analysis. It was shown that a significant heavier band of Ub was pulled down by pFlag-β-TrCP in the cells co-expressing NS1 with pFlag-β-TrCP and pHA-Ub (Figure 3D). The results showed that NS1 could enhance the promotion of β-TrCP degradation through ubiquitination.

### 3.5. β-TrCP Affects IAV Replication

**Figure 4:**
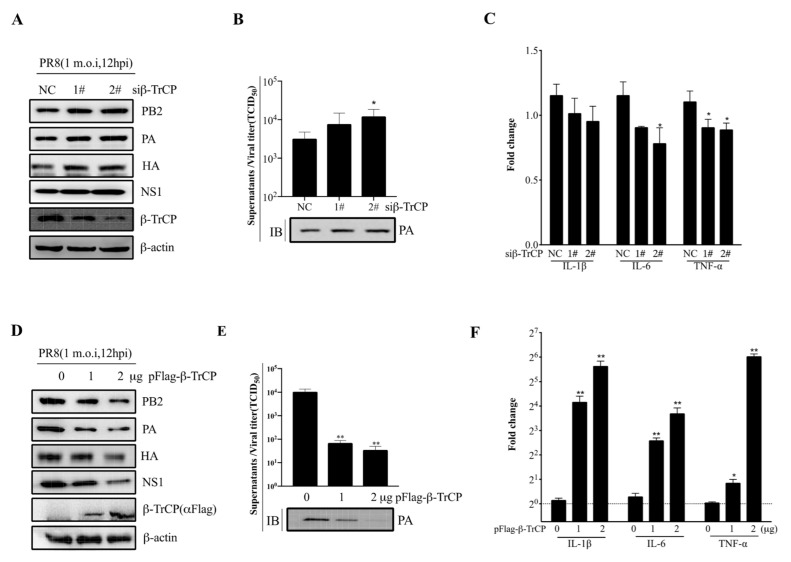
β-TrCP affects influenza A virus replication. The effect of β-TrCP knock-down or overexpression on PR8 replication in 293T cells. Briefly, 293T cells were transfected with β-TrCP small interfering RNA (siβ-TrCP) or pFlag-β-TrCP eukaryotic plasmid for 48 h followed by PR8 virus infection with a dose of 1 m.o.i for 12 h, and the cellular lysates were harvested and immunoblotted with PB2, PA, HA, NS1, and β-TrCP (**A**) or Flag (**D**) antibodies, and β-actin was used as a loading control. The cellular supernatants were titrated in MDCK cells for the TCID_50_ assay and the protein level of PA was detected by immunoblot analysis (**B**,**E**). The transcriptional changes in IL-1β, IL-6, and TNF-α mRNAs were determined by qRT-PCR (**C**,**F**). The data represent the mean values ± SD from three independent experiments in triplicate. *: *p* < 0.05, **: *p* < 0.01.

To test whether β-TrCP affects IAV replication in mammalian cells, HEK-293T cells were either transfected with small interfering RNA (siRNA) (siβ-TrCP-1# and siβ-TrCP-2#,10 nM) or pFlag-β-TrCP(0,1 and 2μg) eukaryotic plasmid for 48 h followed by infection of these cells with PR8 at 1 m.o.i for an additional 12 h, respectively. The cell lysates and supernatants were collected separately for immunoblot analysis and the virus titration assay. It was shown that the expression of β-TrCP was successfully knocked-down in the cells treated with siβ-TrCP, and the expression of viral protein PB2, PA, HA, and NS1 was increased (Figure 4A). Meanwhile, in the cellular supernatants, the replication of PR8 virus was increased in β-TrCP-silenced 293T cells, the virus titration assay was measured by the TCID_50_ assay, and the level of PA protein served as the indicator in immunoblot analysis (Figure 4B). In contrast, overexpression of β-TrCP markedly reduced the expression of viral protein PB2, PA, HA, and NS1 in HEK-293T cells (Figure 4D). At the same time, in the cellular supernatants, the replication of PR8 virus was significantly inhibited by overexpression of β-TrCP (Figure 4E). In more detail, compared to the 0 μg β-TrCP group, the virus titer was reduced 151 times in the 1 μg β-TrCP group and 300 times in the 2 μg β-TrCP group. Moreover, the mRNA expression level of the proinflammatory cytokines, IL-1β, IL-6, and TNF-α were determined by the qPCR assay, and our data showed that the IL-1β, IL-6, and TNF-α mRNA levels were significantly increased in β-TrCP-over-expressed 293T cells (Figure 4F) but only slightly decreased in β-TrCP-silenced 293T cells (Figure 4C). These results suggest that β-TrCP may play an essential role in the regulation of virus replication and the inflammatory response.

## 4. Discussion

Influenza A virus infection still poses a global threat to public health, which causes about 650,000 human deaths annually [28,29]. Several studies have pointed out that deaths caused by highly pathogenic influenza are mostly linked to the presence of the so-called cytokine storm induced by the virus [30,31,32]. Excessive production of pro-inflammatory cytokines leads to ARDS aggravation and widespread tissue damage, resulting in multi-organ failure and death.

IAV NS1 serves as the main viral protein that contracts the antiviral defenses of the host cells. Previous studies demonstrated that IAV employed NS1 to interfere with the RIG-I signaling pathway to escape the host’s innate immune response [33,34,35]. NF-κB is one of the well-studied nuclear transcriptional factors that regulates the transcription of many inflammatory factors. The critical point of this pathway is to induce transcription, but it needs to overcome a significant obstacle, a set of inhibitors (IκBs) that bind NF-κB and prohibit either the nuclear entry or the DNA binding of the transcriptional factor. Thus, activation of the IκB kinase (IKK) and degradation of the phosphorylated inhibitors were required for NF-κB activation. IKK activation and IκB degradation involve different ubiquitination modes, and the latter is mediated by a specific E3 ubiquitin ligase SCF^β-TrCP^.

Interestingly, in our studies, the level of β-TrCP was significantly downregulated in mammalian cells infected with IAV virus (Figure 1C,D and Figure 2). Meanwhile, the mRNA expression level of proinflammatory cytokines, IL-1β, IL-6, and TNF-α were remarkably reduced at 12–24 hpi after PR8 infection (Figure 1A,B), which showed a high association with the decrease in β-TrCP. Our data suggest that β-TrCP may play a key role in the regulation of IAV replication and pathogenesis.

To illustrate our speculation that β-TrCP might interact with IAV proteins to regulate virus replication, we firstly performed the co-IP assay with pFlag-β-TrCP transfection and PR8 virus infection 293T cells, and the data showed that only NS1 associates with β-TrCP (Figure 3A). In addition, we examined the interaction of maCa04 (H1N1) NS1 protein with β-TrCP and found that both maCa04 and PR8 NS1 proteins associated with β-TrCP (Figure S1), suggesting that IAV NS1 protein can be physically associated with β-TrCP. So far, IAV NS1 has been confirmed by many studies to participate in regulating inflammatory response [36,37,38]. Gao et al. reported that NS1 explicitly inhibits IKK-mediated NF-κB activation by physically interacting with IKK. Thus, NS1 diminishes antiviral immune responses and enhances viral pathogenesis [39].

Subsequently, β-TrCP was detected in 293T cells with ectopic expression of PR8 NS1 proteins, and the level of β-TrCP decreased in a dose-dependent manner of NS1 expression (Figure 3B,C). Then, we found that the ability of NS1 to decrease the level of β-TrCP was blocked by treatment with MG132 (5 μM) proteasome inhibitor (Figure 3B,C), which suggests that NS1 targets β-TrCP for proteasomal degradation. To investigate whether NS1 promotes β-TrCP degradation via ubiquitination, pFlag-β-TrCP and pHA-Ub were co-expressed in 293T cells with Myc-tagged NS1 (PR8) (Figure 3D). We showed that pFlag-β-TrCP was detected along with slower migrating bands by anti-Flag antibody in 293T cells expressing NS1 (Figure 3D, compare lane 3 with lanes 1 and 2). We also showed that the slow migrating bands were recognized by anti-ubiquitin antibodies (Figure 3D, Ub panel). This finding indicates NS1 promotes β-TrCP degradation via ubiquitination.

To better explain the relationship between β-TrCP and IAV replication, we found that the replication of PR8 virus was significantly inhibited in β-TrCP overexpression (Figure 4D,E), and the mRNA expression levels of proinflammatory cytokines, IL-1β, IL-6, and TNF-α were obviously increased (Figure 4F). In contrast, the replication of PR8 virus was increased in β-TrCP-silenced 293T cells (Figure 4A,B). Taken together, our findings suggested that β-TrCP plays an essential role in the regulation of virus replication and the inflammatory response, and its functional role might be associated with IAV proteins.

In conclusion, we investigated the relationship between the influenza virus NS1 protein and host β-TrCP protein. Our data show that β-TrCP inhibits viral replication by targeting viral non-structural protein 1 (NS1), in addition to which the NS1 protein in turn inhibits β-TrCP levels. Our results provide a novel mechanism for influenza to modulate its immune response during infection, and β-TrCP may be a novel target against influenza viruses.

## Data Availability

Not applicable.

## Supplementary Materials

The following supporting information can be downloaded at: https://www.mdpi.com/article/10.3390/v14112426/s1, Figure S1: Host protein β-TrCP associates with Influenza A virus NS1 protein; Figure S2: β-TrCP affects Influenza A virus replication; Figure S3: NS1 interaction with cellular β-TrCP.
