# Supplementary material for: Inter-Fighting between Influenza A Virus NS1 and β-TrCP: A Novel Mechanism of Anti-Influenza Virus
Source: Viruses. 2022 Oct 31;14(11):2426. doi: 10.3390/v14112426 (PMC9699209; doi:10.3390/v14112426)

Figure S1. Host protein β-TrCP associates with Influenza A virus NS1 protein
293T cells were transfected with Flag-β-TrCP and HA-NS1(PR8) for 48 hours and then immunoprecipitated with anti-Flag (**A**) and anti HA (**B**), then immunoblotted with anti-Flag and anti-HA antibodies. 293T cells were transfected Flag-β-TrCP with HA-NS1 of PR8 and maCa04, the cell lysates were immunoprecipitated with anti-HA and immunoblotted with anti-Flag and anti-HA (**C**).

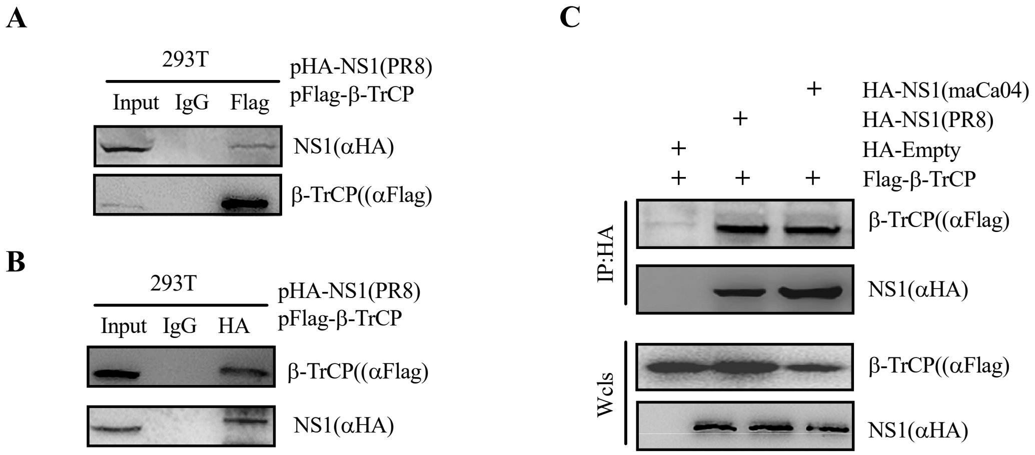

Figure S2. β-TrCP affects Influenza A virus replication. The effect of β-TrCP knock-down or overexpress on PR8 replication in 293T and A549 cells. Briefly, cells were transfected with β-TrCP small interfering RNA (siβ-TrCP) or pFlag-β-TrCP eukaryotic plasmid for 48 hours and then followed by PR8 virus infection with a dose of 1 m.o.i for 12 hours, and the celllуar lysates were harvested and immunoblotted with PB2, PA, HA, NS1 and β-TrCP (**A** and **C**) or and Flag (**B** and **D**) antibodies, and β-actin as a loading control.

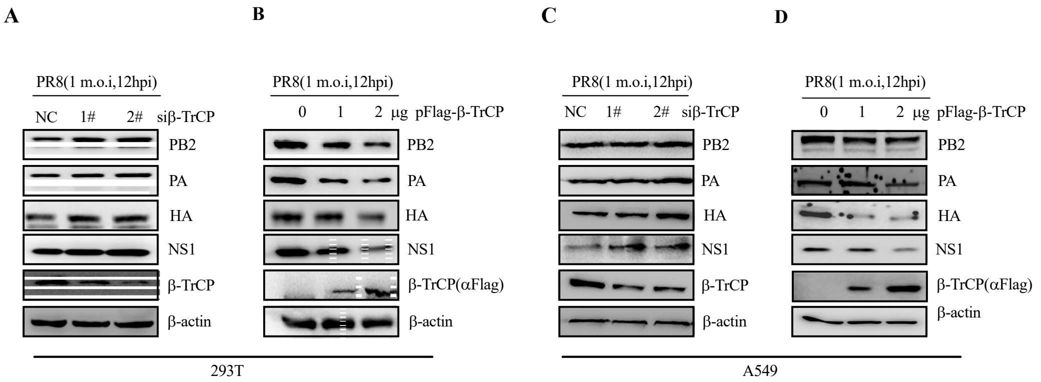

Figure S3. NS1 interaction with cellular β-TrCP. 293T cells were infected with PR8 virus at 1 m.o.i for 12 hours, then the cell lysates were harvested and immunoprecipitated with anti-β-TrCP and immunoblotted with anti-β-TrCP and NS1.

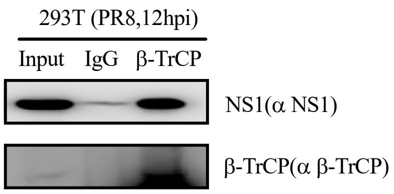

Supplement: Supplementary file 1 [file viruses-14-02426-s001.zip › viruses-2012554-supplementary.pdf]
